# Artificial intelligence manages congenital cataract with individualized prediction and telehealth computing

**DOI:** 10.1038/s41746-020-00319-x

**Published:** 2020-08-28

**Authors:** Erping Long, Jingjing Chen, Xiaohang Wu, Zhenzhen Liu, Liming Wang, Jiewei Jiang, Wangting Li, Yi Zhu, Chuan Chen, Zhuoling Lin, Jing Li, Xiaoyan Li, Hui Chen, Chong Guo, Lanqin Zhao, Daoyao Nie, Xinhua Liu, Xin Liu, Zhe Dong, Bo Yun, Wenbin Wei, Fan Xu, Jian Lv, Min Li, Shiqi Ling, Lei Zhong, Junhong Chen, Qishan Zheng, Li Zhang, Yi Xiang, Gang Tan, Kai Huang, Yifan Xiang, Duoru Lin, Xulin Zhang, Meimei Dongye, Dongni Wang, Weirong Chen, Xiyang Liu, Haotian Lin, Yizhi Liu

**Affiliations:** 1 State Key Laboratory of Ophthalmology, Zhongshan Ophthalmic Center, Sun Yat-sen University, Guangzhou, China; 2 School of Computer Science and Technology, Xidian University, Xi’an, China; 3 School of Software, Xidian University, Xi’an, China; 4 School of Electronics Engineering, Xi’an University of Posts and Telecommunications, Xi’an, China; 5 Department of Molecular and Cellular Pharmacology, University of Miami Miller School of Medicine, Miami, Florida USA; 6 Shenzhen Eye Hospital, Shenzhen Key Laboratory of Ophthalmology, Shenzhen University School of Medicine, Shenzhen, China; 7 Beijing Tongren Eye Center, Beijing Tongren Hospital, Capital Medical University, Beijing, China; 8 Department of Ophthalmology, People’s Hospital of Guangxi Zhuang Autonomous Region, Nanning, Guangxi China; 9 Department of Ophthalmology, The Third Affiliated Hospital of Sun Yat-Sen University, Guangzhou, China; 10 Puning People’s Hospital, Southern Medical University, Jieyang, China; 11 Department of Ophthalmology, The Central Hospital of Wuhan, Tongji Medical College, Huazhong University of Science and Technology, Wuhan, China; 12 The First Affiliated Hospital of University of South China, Hengyang, China; 13 School of Data and Computer Science, Sun Yat-sen University, Guangzhou, 510060 China

**Keywords:** Lens diseases, Translational research, Computer science, Health care economics

## Abstract

A challenge of chronic diseases that remains to be solved is how to liberate patients and medical resources from the burdens of long-term monitoring and periodic visits. Precise management based on artificial intelligence (AI) holds great promise; however, a clinical application that fully integrates prediction and telehealth computing has not been achieved, and further efforts are required to validate its real-world benefits. Taking congenital cataract as a representative, we used Bayesian and deep-learning algorithms to create CC-Guardian, an AI agent that incorporates individualized prediction and scheduling, and intelligent telehealth follow-up computing. Our agent exhibits high sensitivity and specificity in both internal and multi-resource validation. We integrate our agent with a web-based smartphone app and prototype a prediction-telehealth cloud platform to support our intelligent follow-up system. We then conduct a retrospective self-controlled test validating that our system not only accurately detects and addresses complications at earlier stages, but also reduces the socioeconomic burdens compared to conventional methods. This study represents a pioneering step in applying AI to achieve real medical benefits and demonstrates a novel strategy for the effective management of chronic diseases.

## Introduction

A medical revolution driven by artificial intelligence (AI) is expected in the near future^1–3^. Individualized prediction using machine learning promises to be transformative and indispensable for complex medical situations^4^, including nephrology^5^, cardiology^6^, and ophthalmology^7^. Moreover, intelligent telehealth computing has shown great potential in enabling cost-effective applications of medical AI^8^. Although the promise of these technologies is broad, a full integration of prediction and telehealth has not been achieved, and the actual benefits of AI regarding healthcare quality and socioeconomic burden remain to be validated.

Chronic diseases and conditions are the leading causes of death and disability worldwide^9^. Follow-up management of chronic-disease patients remains one of the most intractable healthcare problems, and solutions are urgently needed^10^. Presently, conventional follow-up plans scheduled by clinicians are one-size-fits-all and are primarily based on personal experience or limited clinical evidence, resulting in the delayed detection of complications^11^. Furthermore, among developing countries, only a few specialized care centers are capable of effective examinations and accurate interventions^12,13^. The sparse distribution of these specialized centers creates significant difficulty and economic pressure for patients, resulting in low follow-up rates^14^. Therefore, a new strategy for precise and effective follow-up management leveraging AI is highly desirable.

The study focuses on the follow-up management of patients with congenital cataract (CC), a typical chronic condition characterized by high long-term risk of two main complications: high intraocular pressure (IOP)^15^ and visual axis opacification (VAO)^16^. Rigorous follow-up care and timely intervention are necessary to prevent the irreversible and permanent loss of vision caused by these complications^17^. Therefore, CC is an ideal test case for exploring follow-up management strategies for chronic conditions.

To explore the feasibility of applying AI to improve the quality of follow-up care, we applied Bayesian and deep-learning algorithms to create CC-Guardian, an intelligent agent that consists of three functional modules: (i) a prediction module that identifies potential high-risk CC patients who are likely to suffer complications, (ii) a dispatching module that schedules individual follow-up based on the prediction results, and (iii) a telehealth module that makes intervention decisions in each follow-up examination. We verified CC-Guardian’s performance using internal and multi-resource validation. Using CC-Guardian, we implemented a web-based smartphone app and proposed an operating mechanism for our intelligent follow-up system. We conducted a retrospective self-controlled test to investigate the real-world efficiency of our system in terms of complication prediction, telehealth detection, and cost-efficient benefits. More broadly, this study takes a pioneering step in translating AI into actual medical benefits and provides a novel approach to the effective management of chronic conditions.

## Results

### Dataset collection and preparation for agent training

**Fig. 1:**
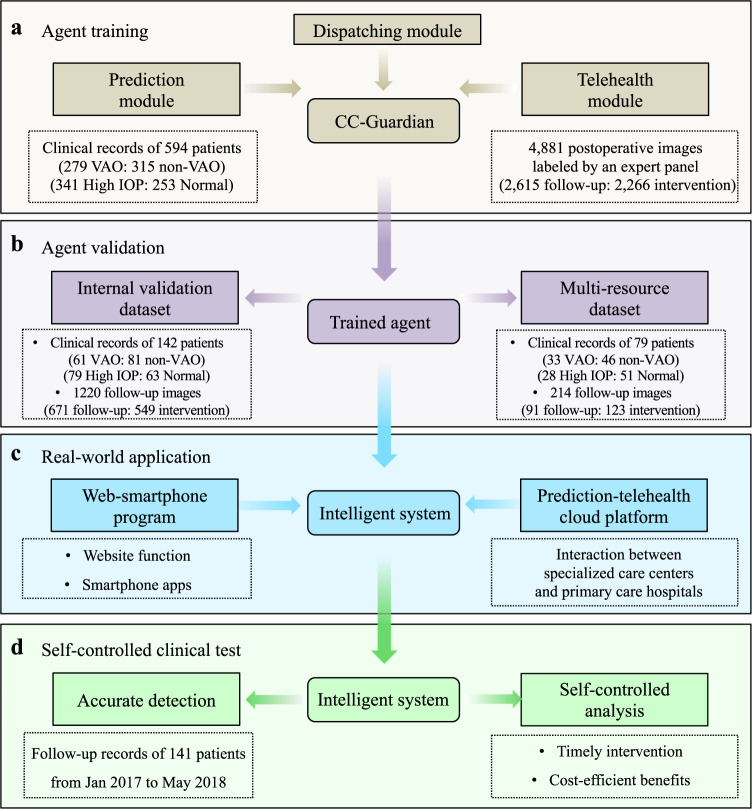
Study pipeline for agent training, validation, application, and testing. **a** Multidimensional clinical records of 594 congenital cataract patients were collected for prediction module training (279 VAO: 315 non-VAO; 341 high IOP: 253 normal). For telehealth module training, a total of 4881 postoperative retro-illumination images were obtained (2615 follow-up: 2266 intervention). Each image was independently described and labeled by an expert panel. **b** Two datasets were used for the validation of the trained agent, including internal validation dataset (clinical records of 142 patients, and 1220 follow-up images) and multi-resource dataset (clinical records of 79 patients, 214 follow-up images). **c** A web-based smartphone app was implemented, and a prediction-telehealth cloud platform was prototyped for the clinical application of our intelligent follow-up system. **d** A retrospective self-controlled test was conducted to investigate the real-world efficiency of our follow-up system in complication prediction, telehealth detection, and cost-effect benefit. VAO visual axis opacification, IOP intraocular pressure.

The pipeline for our study is shown in Fig. 1. The training dataset included clinical records of 594 CC patients before and after surgery, and 4881 of their follow-up images from January 2011 to December 2016. The eligible criteria of patients included diagnosis of CC, informed consent, and completed records of baseline information, lesion condition, comorbidities, surgical procedures, and complications in the first follow-up year. The eligible images for training were defined as the anterior segment images covering the posterior lens capsule by retro-illumination using slit-lamp photography. All these records were derived from routine examinations at the Childhood Cataract Program of the Chinese Ministry of Health (CCPMOH)^18^.

### Functional architecture of CC-Guardian

**Fig. 2:**
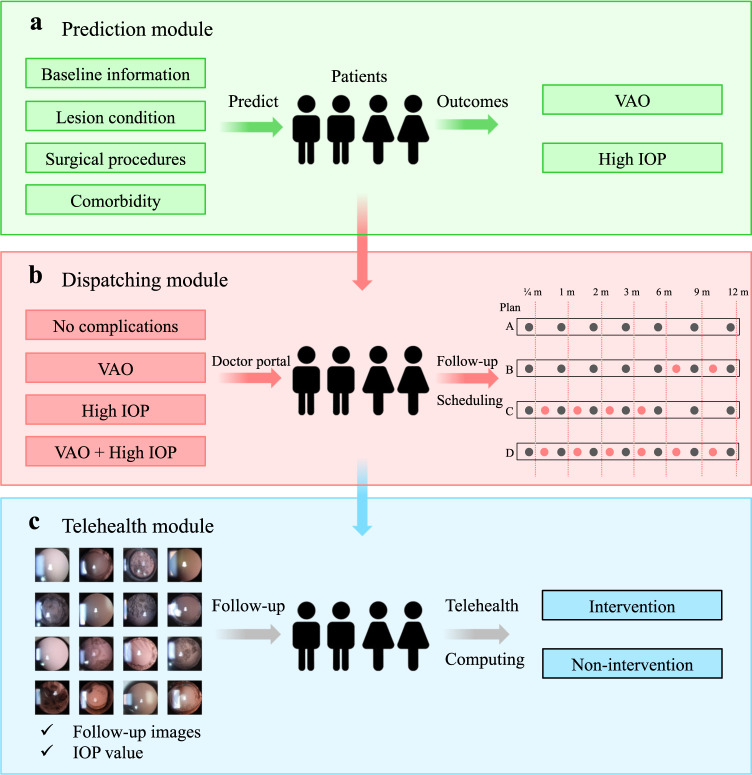
Functional architecture of CC-Guardian. **a** The prediction module is trained to identify high-risk patients likely to suffer complications (the occurrence of VAO, happen or not and the occurrence of high IOP, happen or not). **b** The dispatching module is responsible for scheduling individual follow-up based on the prediction module results. **c** In the telehealth module, a clinical decision regarding further treatment (intervention or continued follow-up) is made after each telehealth examination based on follow-up images and IOP values. VAO visual axis opacification, IOP intraocular pressure, m months.

The CC-Guardian incorporates three functional modules: a prediction module designed to identify high-risk patients who are likely to suffer complications (Fig. 2a), a dispatching module responsible for scheduling individual follow-up visits (depending on the results of the prediction module) (Fig. 2b), and a telehealth module that makes a clinical decision regarding additional treatment (intervention or continued follow-up) in each follow-up examination using telehealth computing (Fig. 2c).

### Naive Bayes algorithm for prediction module

We selected 12 variables that both can be easily collected in routine clinical practice and were reported to be associated with complications after surgery^16,17^ as the inputs for the prediction module. These 12 variables can be generally grouped into four categories: (1) baseline information, (2) lesion condition, (3) comorbidities, and (4) surgical procedures (“Methods”).

To find the optimal algorithm, we first performed a comparison among naive Bayes and two classic machine learning algorithms (random forest and neural network, “Methods”). Based on the average performance of the 5-fold cross-validation by the training set of 594 CC patients, the naive Bayes algorithm provided comparative performance with random forest and neural network algorithms (Table 1). We finally selected the naive Bayes algorithm due to its intrinsic simplicity^19^, which can improve the processing speed and saving computing resource in real-world application. After selecting the naive Bayes, a complete algorithm was then trained on the entire dataset of 594 CC patients prior to later validations.

**Table 1:** Performance of methodological comparison among algorithms.

	Outcome	Accuracy	Sensitivity	Specificity
Naïve Bayes	VAO	0.966 (0.916–0.991)	0.964 (0.875–0.996)	0.969 (0.892–0.996)
	High IOP	0.975 (0.928–0.995)	0.972 (0.902–0.997)	0.979 (0.889–0.999)
Random forest	VAO	0.950 (0.894–0.981)	0.946 (0.849–0.989)	0.953 (0.869–0.990)
	High IOP	0.941 (0.883–0.976)	0.944 (0.862–0.984)	0.938 (0.828–0.987)
Neural network	VAO	0.950 (0.894–0.981)	0.909 (0.801–0.970)	0.984 (0.916–0.999)
	High IOP	0.933 (0.872–0.971)	0.986 (0.924–0.999)	0.854 (0.722–0.939)

*VAO* visual axis opacification, *IOP* intraocular pressure, *accuracy* (TP + TN)/(TP + TN + FP + FN), *sensitivity* TP/(TP + FN), *specificity* TN/(TN + FP), *TP* true positive, *TN* true negative, *FP* false positive, *FN* false negative, *CI* confidence interval.

A methodological comparison among naive Bayes, random forest, and neural network was performed using the average performance of the 5-fold cross-validation by the training set of 594 patients.

The normalized contributions of all 12 input variables to predict VAO (from 0.01 to 0.2, Supplementary Fig. 1a) and high IOP (from 0.03 to 0.15, Supplementary Fig. 1b) were presented, suggesting their substantial importance to outcome. Both the age at surgery (0.07 to VAO and 0.12 to high IOP) and gender (0.03 to VAO and 0.02 to high IOP) do not show any dominant role, indicating that predictive results will not significantly vary from these two variables.

### Follow-up schedules for dispatching module

For patients predicted with no complications, the dispatching module will schedule seven time points (1 week, 1 month, 2 months, 3 months, 6 months, 9 months, and 12 months after surgery), which is the conventional plan in current clinical practice^14^. If the patients were predicted to have VAO, the module will add two time points (7.5 months and 10.5 months) to the conventional plan, since VAO was inclined to happen in the second half year^17,20^. If the patients were predicted to have high IOP, the module will add three time points (2.5 weeks, 2.5 months and 4.5 months) to the conventional plan, since high IOP was inclined to happen in the second half year^16,20^.

### Deep residual network for telehealth module

A total of 4881 postoperative retro-illumination images were included as the training set for telehealth module. These 4881 images were collected from 2175 CC patients. Images from the same child were taken at different follow-up visits. Each image was independently labeled (intervention or continued follow-up) by two licensed ophthalmologists, and a third ophthalmologist was consulted when disagreements occurred. Then, a senior ophthalmologist with over 20 years of clinical CC experience verified the labels for each image. The ophthalmologists were blind and had no access to the outcome classifications. A stacked 101-layer residual network^21^ was utilized for telehealth module training and classification (“Methods”). The first 100 layers were used to extract multidimensional, high-level features from the input images and a Softmax classifier was applied to the last layer. The architecture of the deep residual network is provided in Supplementary Fig. 2 in the form of a diagram that highlights the arrangement of each layer.

### Internal validation

A randomly selected dataset derived from the original CCPMOH records prior to training was used to validate CC-Guardian, which included clinical records of 142 patients (61 VAO, 81 non-VAO; 79 high-IOP, 63 normal) and 1220 follow-up images (671 follow-up, 549 intervention). The 1,220 images were collected from 507 CC patients. This dataset did not overlap with the CCPMOH data for agent training. The trained deep-learning model was frozen prior to any validations. The deep-learning predictions with time stamps were verified and saved by an individual who was blind to the expert-panel labels, thus precluding information leaking or double-dipping when comparing the prediction results with the expert-panel labels. After internal validation, the models trained on all of the training sets were used for following validation.

**Fig. 3:**
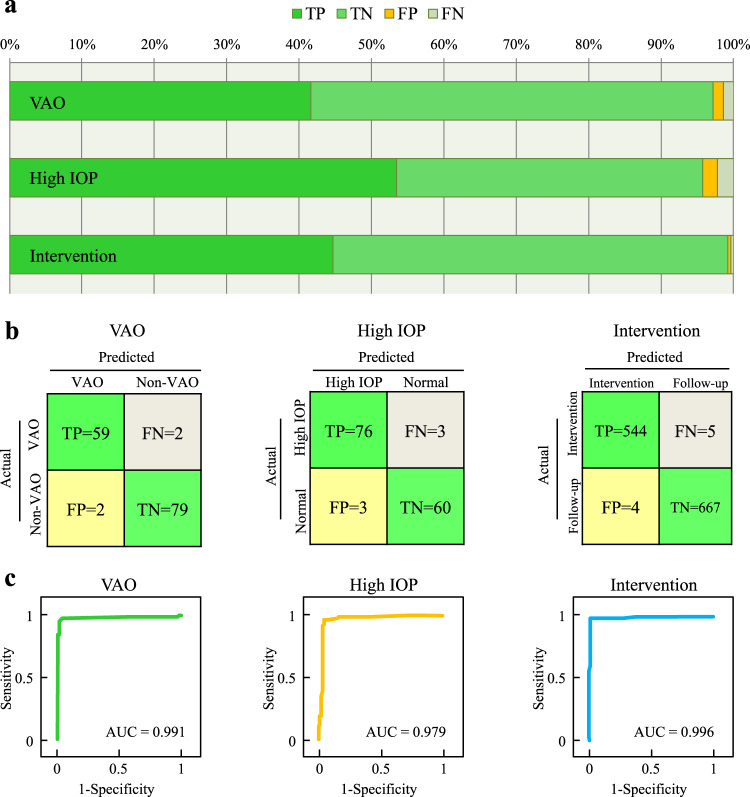
Highly accurate performance of CC-Guardian in internal validation. **a** Using the prediction module, our agent predicted VAO with 96.7% sensitivity and 97.5% specificity, and high IOP with 96.2% sensitivity and 95.2% specificity in internal validation. Using the telehealth module, our agent provided intervention suggestions with 99.1% sensitivity and 99.4% specificity. **b** Confusion matrices for agent validation. **c** Our agent had AUCs of 0.991, 0.979, and 0.996 for detecting VAO, high IOP, and intervention, respectively, in internal validation. AUC area under curve, TP true positive, TN true negative, FP false positive, FN false negative.

Using the prediction module, our agent predicted VAO with sensitivity of 0.967, specificity of 0.975, and area under curve (AUC) of 0.991; and high IOP with sensitivity of 0.962, specificity of 0.952, and AUC of 0.979. By virtue of its telehealth module, CC-Guardian provides intervention decisions with sensitivity of 0.991, specificity of 0.994, and AUC of 0.996. The detailed distributions for the accurate and mistaken detections are presented in Fig. 3a. Confusion matrices and receiver operating characteristic (ROC) curves are shown in Fig. 3b, c, respectively.

### Multi-resource validation

The multi-resource dataset had two parts. The first part consisted of clinical records of 79 patients (33 VAO, 46 non-VAO; 28 high-IOP, 51 normal) collected from 7 centers, which were used for validating prediction module. Seven centers included Shenzhen Eye Hospital, Beijing Tongren Hospital, People’s Hospital of Guangxi Zhuang Autonomous Region, the Third Affiliated Hospital of Sun Yat-sen University, Puning People’s Hospital, The Central Hospital of Wuhan, and the First Affiliated Hospital of University of South China. These 7 centers were distributed separately in northern, central, and southern China, and thus collectively composed a representative and independent sample for Chinese population. The eligible criteria were the same as the previous datasets, which were diagnosis of CC, informed consent, and completed records of inputs and outcomes required for prediction module. Detailed description of the datasets collected in each center was summarized in Supplementary Table 1.

The second part contained images collected from heterogeneous open-access databases, which can further validate versatility and generalization of telehealth module. We performed image searches using the Google, Baidu and Bing search engines through June 2016, with a combination of key words (e.g., congenital, infantile, pediatric cataract, follow-up, and retro-illumination) in the form of title words or medical subject headings. Two individuals (E.L. and H.L.) independently completed the searches. In addition, these two individuals cross-checked and confirmed that all the collected cases were retro-illumination images of CC during follow-up management. When discrepancies arose, consensus was achieved after further discussion. All confirmed images (91 follow-up and 123 intervention) were subsequently sent to CC-Guardian for validation.

**Fig. 4:**
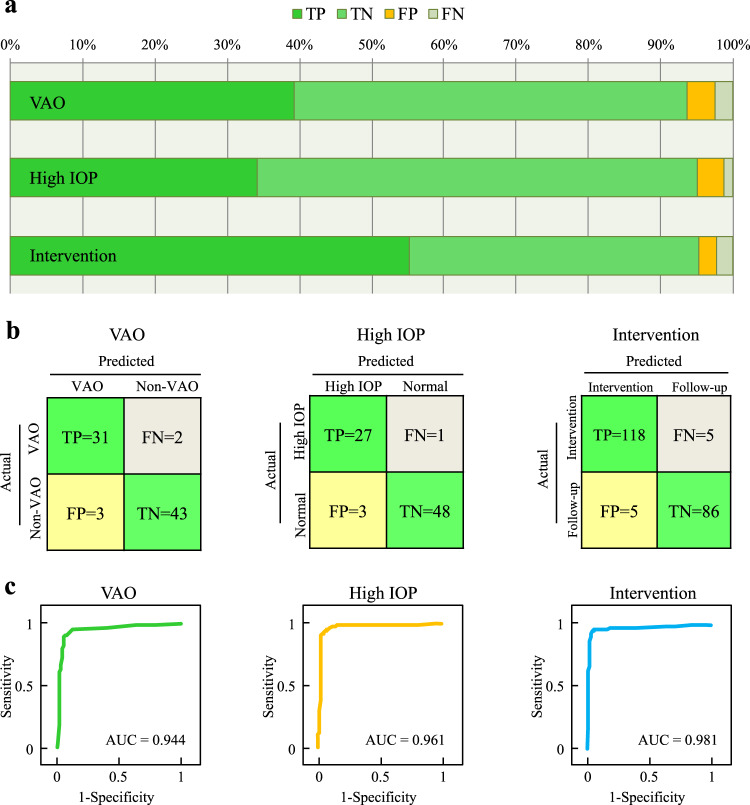
Comparative performance of CC-Guardian in multi-resource validation. **a** Using the prediction module, our agent predicted VAO with 94.0% sensitivity and 93.5% specificity, and high IOP with 96.4% sensitivity and 94.1% specificity for the multi-resource dataset. Using the telehealth module, our agent provided intervention suggestions with 95.9% sensitivity and 94.5% specificity for the multi-resource dataset. **b** Confusion matrices for agent validation. **c** Our agent had AUCs of 0.944, 0.961, and 0.981 for detecting VAO, high IOP, and intervention, respectively, for the multi-resource dataset. AUC area under curve, TP true positive, TN true negative, FP false positive, FN false negative.

The CC-Guardian’s prediction module predicted VAO with sensitivity of 0.940, specificity of 0.935, and area under curve (AUC) of 0.944; and high IOP with sensitivity of 0.964, specificity of 0.941, and AUC of 0.961. The CC-Guardian’s telehealth module provided intervention suggestions with sensitivity of 0.959, specificity of 0.945, and AUC of 0.981, a performance comparable to that obtained from internal validation. The detailed distributions for the accurate and mistaken detections are presented in Fig. 4a. Confusion matrices and ROC curves are shown in Fig. 4b, c, respectively.

### CC-Guardian web-based smartphone app

To establish a telehealth cloud platform for clinical application, we built a CC-Guardian web-based smartphone app. The functions of the website (available at https://www.cc-cruiser.com/cc_guardian) include prediction-metric input, risk-stratification output, follow-up-examination results upload, and intervention decisions reporting. The functions of the smartphone app (available for iOS and Android systems) include a doctor portal (individual follow-up scheduling and updating) and a patient portal (automatic short message service (SMS) and interaction between patients and CCPMOH).

Users login to the website and input the necessary prediction metrics to obtain the risk-stratification output (occurrence of VAO and/or high IOP) (Supplementary Fig. 3a). Doctors can assign an individual follow-up plan using the smartphone app (doctor portal) (Supplementary Fig. 3b). Patients will receive individual follow-up schedules and can check these schedules using the smartphone app (patient portal) (Supplementary Fig. 3c). Interaction between patients and CCPMOH is enabled through either the website or the app. Doctors can also reschedule individual plans if necessary. During the follow-up process, users can upload their follow-up examination results to the website and obtain an intervention decision in a timely manner (Supplementary Fig. 3d). The algorithm embedded in this app is the same as the one in following validations.

### CC-Guardian prediction-telehealth cloud platform

**Fig. 5:**
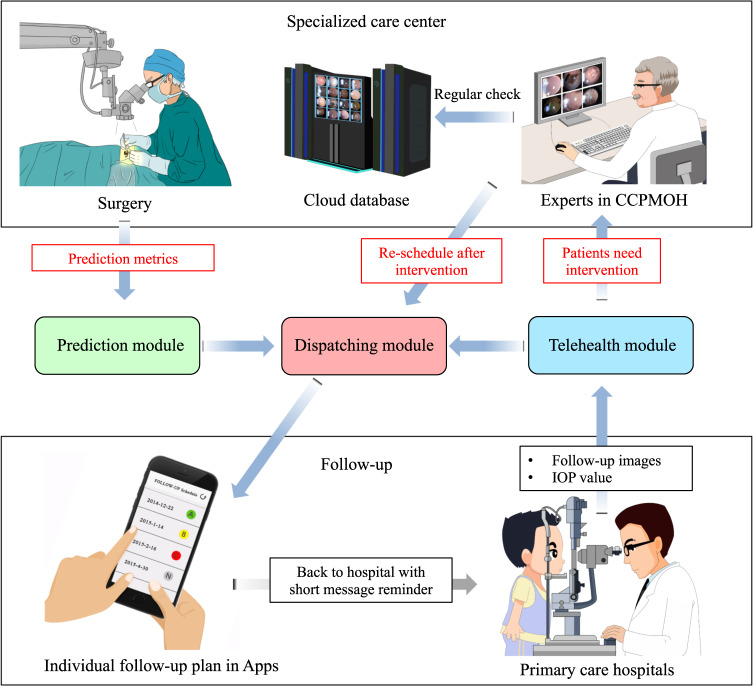
The prediction-telehealth cloud platform. When potential patients register at the specialized care center (CCPMOH), their clinical metrics (valuable for the prediction module) are collected with their permission and immediately uploaded to the CC-Guardian cloud platform for complication prediction. Based on the prediction results, the dispatching module designs an individualized follow-up schedule and sends a short message to notify each corresponding patient in a timely manner. Patients can complete their regular follow-up in primary care hospitals and upload their examination results to the web-based telehealth module. If the telehealth module recommends intervention, the fast-track notification system is triggered, and an emergency notification is sent to doctors at the specialized care center (CCPMOH) for immediate confirmation. These patients are informed that they should undergo intervention to manage complications after confirmation.

A compatible mechanism to promote the real-world clinical application of CC-Guardian to the follow-up management is highly desirable. Therefore, we created a prediction-telehealth cloud platform for the CC-Guardian smartphone app. As shown in Fig. 5, when a potential patient registers at the specialized care center (CCPMOH), their clinical metrics (valuable for the prediction module) are collected with their permission and immediately sent to the CC-Guardian website for complication prediction. Based on the prediction results, the dispatching module individually determines a follow-up schedule and sends an SMS to remind the patient in a timely manner. Patients can complete their regular follow-up examinations in primary care hospitals (telehealth follow-up examination) and upload their examination results to the web-based telehealth module. If the telehealth module recommends intervention, a fast-track notification system is triggered, and an emergency notification is sent to doctors at CCPMOH for immediate confirmation. Such patients will be informed that they should undergo intervention to manage their complications. Instructions for using the prediction-telehealth cloud platform are provided in Supplementary Video 1.

### Retrospective self-controlled test

**Fig. 6:**
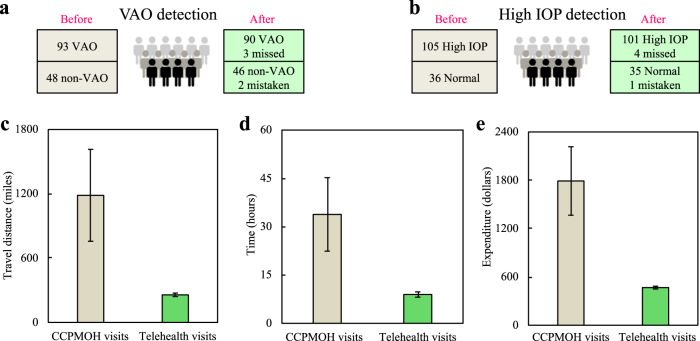
Real-world efficiency in retrospective self-controlled test. Error bars presented the standard deviation. **a**, **b** The longitudinal follow-up records of 141 CC patients (987 follow-up visits to CCPMOH) were retrospectively used for real-world testing. Before using CC-Guardian, a total of 93 patients underwent VAO and 105 patients suffered from High IOP. After using our system, 90 cases of VAO (90/93, 96.8%) and 101 cases of high IOP (101/105, 96.2%) are successfully predicted. **c**–**e** Each family would have saved an average travel distance of 928.6 miles (1185.4 vs. 256.8 miles, *P* < 0.001), an average time of 24.9 h (33.8 vs. 8.9 h, *P* < 0.001), and an average expenditure of $1324.1 ($1791.4 vs. $467.3, *P* < 0.001) per year. VAO visual axis opacification, IOP intraocular pressure.

To further investigate the real-world efficiency of our system, we retrospectively used longitudinal follow-up records of 141 CC patients from CCPMOH between January of 2016 and May of 2017 (“Methods”). Before using our system, these 141 CC patients had 987 distant follow-up visits to CCPMOH, including 93 patients who underwent VAO and 105 patients who suffered from high IOP (Fig. 6a, b).

After applying our system, 90 cases of VAO (90/93, 96.8%) and 101 cases of high IOP (101/105, 96.2%) were successfully predicted (Fig. 6a, b). A total of 73 patients (73/90, 81.1%) would have benefited from earlier VAO detection (2759 risk days in total, 37.8 risk days earlier per person on average, median 45.0 risk days) and 89 patients (89/101, 88.1%) would have benefited from earlier high-IOP detection (1709 risk days in total, 19.2 risk days earlier per person on average, median 15.0 risk days).

All 141 patients would have had a total of 1579 telehealth visits rather than 987 distant visits to the CCPMOH, which would have, on average, saved each family significant travel of 928.6 miles per year (1185.4 vs. 256.8 miles, *P* < 0.001, Fig. 6c), time of 24.9 h per year (33.8 vs. 8.9 h, *P* < 0.001, Fig. 6d), and an expenditure of $1324.1 per year, ($1791.4 vs. $467.3, *P* < 0.001, Fig. 6e). These results demonstrate that our system has the potentials to detect and address the complications at an earlier stage, and to reduce the socioeconomic burden faced by patients compared to conventional in-person follow-up.

## Discussion

In this study, we integrated the benefits of individualized prediction and telehealth computing to create an AI agent and introduced a prediction-telehealth cloud platform to support its clinical application to the management of congenital cataract. With high precision as a prerequisite, our system may effectively translate correct predictions and detections into real advancements in treatment timing and significant savings in travel distance, time, and cost.

Earlier diagnosis and timely interventions in response to serious complications can significantly improve healthcare quality^22^. For CC patients whose visual development is in a sensitive stage, even short duration form-deprivation due to VAO will significantly increase the risk of amblyopia^23^. There is a greater risk of glaucoma caused by high IOP, which is one of the leading causes of blindness, progressing rapidly and causing irreversible damage to the optic nerve^24^. Unfortunately, most symptoms of complications (e.g., decreased visual acuity) are insidious and tend to be neglected, which can cause patients to miss the “treatment window” for saving their remaining vision^25^. Therefore, the earlier diagnosis (37.8 risk days per person for VAO and 19.2 risk days per person for high IOP) provided by our system may reduce the risk of permanent visual impairment.

Telehealth computing significantly reduces the travel distance, time, and cost to our patients during follow-up visits (928.6 miles, 24.9 h, and $1324.1 per year for each family), which can substantially increase their compliance. Such benefits will be even greater when considering reduced labor costs for clinicians and patients. Moreover, experts in specialized centers will be free to transfer to more first-visit patients. Therefore, from this perspective, our telehealth pattern will provide substantial impetus for creating a more rational allocation of medical resources and expand the coverage of high-quality medical care.

It should be noted that our previous study has developed an automatic diagnosis system for CC patients^26^. Comparing to our previous work, the current study presented significant advances and novelties. First, our previous study can only be applied in preoperative patients for primary screen and diagnosis. However, the current study focused on follow-up management of CC (high cost of long-term follow-up and delayed detection on postoperative complications), a more general and intractable situation that can be happen in patients with chronic diseases, such as metabolic and cardiovascular diseases. Second, our previous work only analyzed 886 images using a 7-layer convolution network. In comparison, the current work innovatively integrated Bayesian and 101-layer deep residual network to take the advantage of both structured clinical records and 4886 postoperative images, which finally achieved fully automatic flow of prediction and telehealth. Third, although both our previous and current works have achieved highly accurate performance in their specific clinical scenarios, the current work further conducted a retrospective self-controlled test to articulate the exact benefits that our system can provide for patients in real-world settings. These evidence address a matter of greatest concern to both clinicians and patients and provide the direct implications and merit to routine clinical practice.

This study has certain limitations. First, the multi-resource dataset for validating prediction module is relatively small and limited to the Chinese population, due to difficulties for assessing clinical records from centers in other countries^27^ and limited cohorts tracking congenital cataract worldwide^28^. It is also essential to validate our system using datasets in other ethnic population or other countries in the future to achieve global generalization, as previously achieved in diabetic retinopathy^29^. Second, it should be emphasized that the application of our system is limited to CC patients. For other chronic conditions like hypertension and diabetes, it is expected that machine learning can be applied to efficiently manage the ketoacidosis or stroke by prediction-telehealth approach in resource-intensive situations. Third, images used to train the telehealth module are acquired through slit-lamp photography. Therefore, assistances (e.g., strap^30^) would be required for the uncooperative infants during the photography process. Fourth, the duration of self-controlled test is relatively short (one and four months). The records after May 2018 are not available for this test because of the incomplete 1-year follow-up records when we submitted this work. Fourth, the complications’ prevalence in our study is higher than those reported by previous studies^31–33^. It may be attributed to difference of measurement and definition, or “severe patient effects” (that is, infants with complications have better compliance and thus are more likely to have complete follow-up records). We further estimated that such prevalence difference will not influence the results on advance detection timing, savings of travel distance, time and expenditure (Supplementary Information, Supplementary Table 2). Fifth, it should be noted that we cannot quantify the effects of earlier detection on improved outcomes due to retrospective design. It is necessary to conduct prospective studies to further investigate the agent’s robustness in performance and benefits in real-world setting.

In the future, the performance of CC-Guardian will be enhanced though the collection of larger datasets using our platform. Furthermore, CC-Guardian can be combined with wearable devices (e.g., smartphone-based slit-lamp photography) to implement real-time eye condition monitoring, which will provide a foundation for the development of a home-based telehealth platform.

## Methods

### Predictive indicators and outcomes for prediction module

The baseline information included gender, age at surgery, and laterality. The lesion condition included area, density, and location. The area was defined as “extensive” when the opacity covered more than 50% of the pupillary area; otherwise, it was “limited”. The density was defined as “dense” when the opacity fully disrupted viewing of the fundus; otherwise, it was “non-dense”. The location was defined as “central” when the opacity fully covered the visual-axis area; otherwise, it was “peripheral”. For comorbidities, nystagmus, microphthalmia, microcornea, and persistent hyperplastic primary vitreous were defined according to the 11th Revision of the International Classification of Diseases (ICD-11) Beta Draft^34^. For surgical procedures, three standardized cataract extraction methods were classified, including lens aspiration (I/A), I/A with posterior continuous curvilinear capsulorhexis (I/A + PCCC), I/A with PCCC and anterior vitrectomy (A-Vit)^20^. Intraocular lens (IOL) implantation was classified as primary or secondary IOL implantation.

These 12 inputs aim to predict two high-risk complications after one-year of follow-up after surgery: (1) the occurrence of VAO (happen or not) and (2) the occurrence of high IOP (happen or not). VAO was defined as lens epithelial cells proliferation that extends into the pupillary area and covers the visual axis. High IOP was defined as IOP > 21 mmHg. Supplementary Table 3 summarizes the distribution of all inputs and outcomes.

### Methodological comparison for prediction module

To find the optimal algorithm, we first performed a comparison among naive Bayes and two classic machine learning algorithms (random forest and neural network). Briefly, we developed the naive Bayes by constructing a scoring function to perform binary classifications^19^. Random forest was developed by constructing a multitude of decision trees at training time and outputting the mode of the classes for classification^7^. Neural network was developed by systematic training of interlinked simple processing elements and artificial neurons for classification^35^. The methodological comparison was conducted using the toolbox in MatLab 2016 (MathWorks, Inc., Natick, MA, USA). The optimal algorithm was selected based on the average performance of the 5-fold cross-validation by the training set of 594 CC patients. After selection, a complete algorithm was then trained on the entire 594 CC patients prior to later validations.

### Deep residual network for telehealth module

A stacked 101-layer residual network^21^ was utilized for telehealth module training and classification. Specifically, several techniques, including convolution, max pooling, residual blocks, batch-normalizing transform^36^, non-saturating rectified linear units, and data augmentation were integrated into this algorithm. The residual block was employed to avoid the degradation, further enhance recognition rates, and accelerate convergence. The entire network included thirty-three residual blocks, each of which consists of three convolutions and a nonlinear transformation function (i.e., the residual blocks together comprise 99 layers). Batch normalization was employed to address the problem of vanishing and exploding gradients to help convergence. Transfer learning technology was also employed by pre-training on the large-scale 1000-class datasets from ImageNet^37^. We then optimized the learning process on our ocular images by transfer learning an existing model that was. All codes were executed in the Convolutional Architecture for Fast Feature Embedding (Caffe) framework running on an Ubuntu 14.04 64 bit operating system and the Compute Unified Device Architecture (CUDA) 8.0^38^.

### Retrospective self-controlled test

To further investigate the real-world efficiency of the prediction-telehealth cloud platform, we retrospectively used longitudinal follow-up records from CCPMOH between January 2017 and May 2018. Informed consent was obtained from all subjects. Among 172 CC patients, 141 of which were finally included because they had complete follow-up records in all visits. Specifically, we included 141 patients’ clinical records (93 VAO, 48 non-VAO; 105 high-IOP, 36 normal) for prediction module testing and 987 images (394 intervention and 593 follow-up) for telehealth module testing. The mean age at surgery of the included patients were 28.32 months, and 74 participants (52.5%) were male. Supplementary Table 4 summarizes the clinical characteristics of all 141 included patients. All images are eligible as the inputs for the telehealth module.

In the self-controlled analysis, we retrospectively investigated the efficiency (advanced complication detection) and cost-efficient benefits (time, travel distance, and expenditure) before and after the use of our system. For complication detection, true prediction with an advanced schedule and accurate detection was considered a timelier intervention, and the time difference of intervention before and after the use of our system was calculated. For example, an individual had no VAO in 9-month visit but had VAO in 12-month visit, which is considered as 90 risk days before using our system. If our system can predict and detect successfully, this individual will have an additional visit at 10.5 month and be detected at this visit, which is considered as 45 risk days (45 risk days in advance).

The cost-efficient benefits were calculated based on the assumption that all follow-up visits using our agent were conducted via telehealth computing. Travel distance savings was defined as the round-trip distance savings between the distance traveled from the patient’s home address to the telehealth site and the distance the patient would have traveled at CCPMOH. Amap 7.8.6 (Alibaba Group, Hangzhou, China) was used to geocode the patients’ addresses, the telehealth sites (hospitals with ophthalmological departments) and CCPMOH and to calculate the travel distances. Distances were calculated by doubling one-way distances and the “quickest route” option was selected rather than the “shortest route” or “straight line” options.

Time savings was defined as the round-trip time savings for travel to the telehealth sites compared to travel to an in-person consultation at CCPMOH. The following travel speeds were used to calculate travel time: highways, 65 miles per hour (mph); major roads, 50 mph; arterial roads, 30 mph; and streets, 20 mph.

Expenditure savings was defined as the round-trip cost savings between the travel costs associated with traveling to the telehealth sites and those associated with traveling to CCPMOH. The estimated cost savings were calculated according to the latest standard of travel and traffic expenses from the Ministry of Finance of the People’s Republic of China in 2016 (file identifier: 2016–71), which reflects the cost of vehicular travel including insurance, fuel, and vehicle maintenance for miles driven.

### Statistical analysis

Due to the skewed distributions of variables difference, Wilcoxon paired test was used to compare the differences of distance, time, and cost before and after the use of CC-Guardian. The Yonden index method was used to decide the optimal operating threshold for calculating the sensitivity and specificity values^39^. The 95% confidence interval of sensitivity, specificity was calculated using binomial proportion confidence intervals. All the statistical tests were two-tailed, and a *P* value below 0.05 was considered statistically significant. All tests were conducted using the statistical package R, version 3.2.4.

### Ethics approval

Our study was approved by the centralized institutional review board (IRB) of Sun Yat-sen University. It was conducted in accordance with the Declaration of Helsinki. All included datasets were anonymized according to the Health Insurance Portability and Accountability Act Safe Harbor guidelines prior to their transfer to the study investigators.

### Reporting summary

Further information on research design is available in the Nature Research Reporting Summary linked to this article.

## Supplementary information

Supplementary Information

Supplementary Movie 1

Reporting Summary

## Data Availability

The authors declare that the main data supporting the results in this study are available within the paper and its Supplementary Information. The datasets generated during the study are available for research purposes from the corresponding authors on reasonable request.

## Code availability

The source code for the AI agent CC-Guardian is available in Github at https://github.com/longerping/cc-guardian.
