# Supplementary material for: Artificial intelligence manages congenital cataract with individualized prediction and telehealth computing
Source: NPJ Digit Med. 2020 Aug 28;3:112. doi: 10.1038/s41746-020-00319-x (PMC7455726; doi:10.1038/s41746-020-00319-x)
Supplement: Supplementary file 1 — Supplementary Information [file 41746_2020_319_MOESM1_ESM.pdf]

Artificial intelligence manages congenital cataract with individualized prediction and telehealth computing

Supplementary Information

Table of Contents

1. Supplementary Figures.…………………………………………………………….....2

2. Supplementary Tables…………………………………………………………...……6

3. Supplementary Information……………………………………………………....….…10

Supplementary Figures

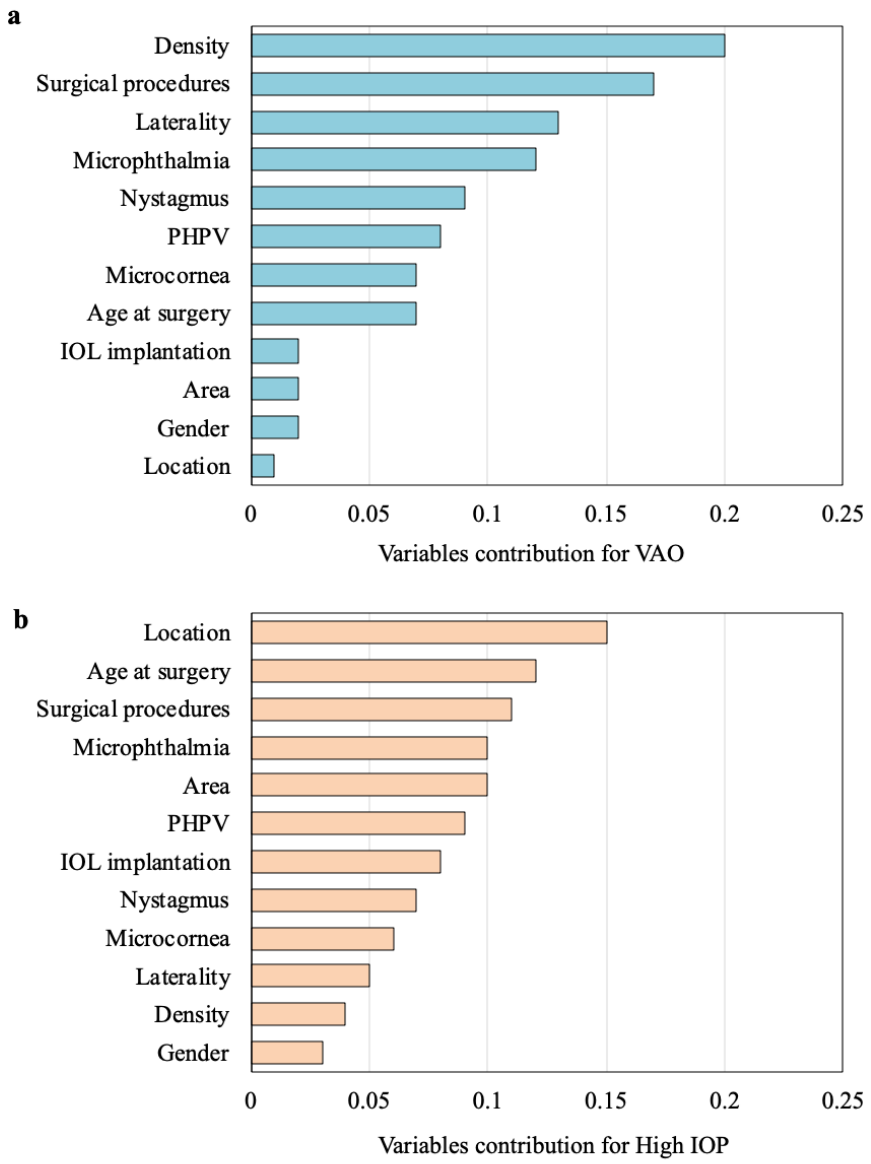

Supplementary Figure 1. Variable contributions in the complete algorithm trained on the entire dataset of 594 patients.

Notes: PHPV = Persistent Hyperplastic Primary Vitreous; VAO = Visual Axis Opacification; IOP = Intraocular Pressure.

a. Normalized variable contributions for predicting the VAO outcome were presented.

b. Normalized variable contributions for predicting the high IOP outcome were presented.

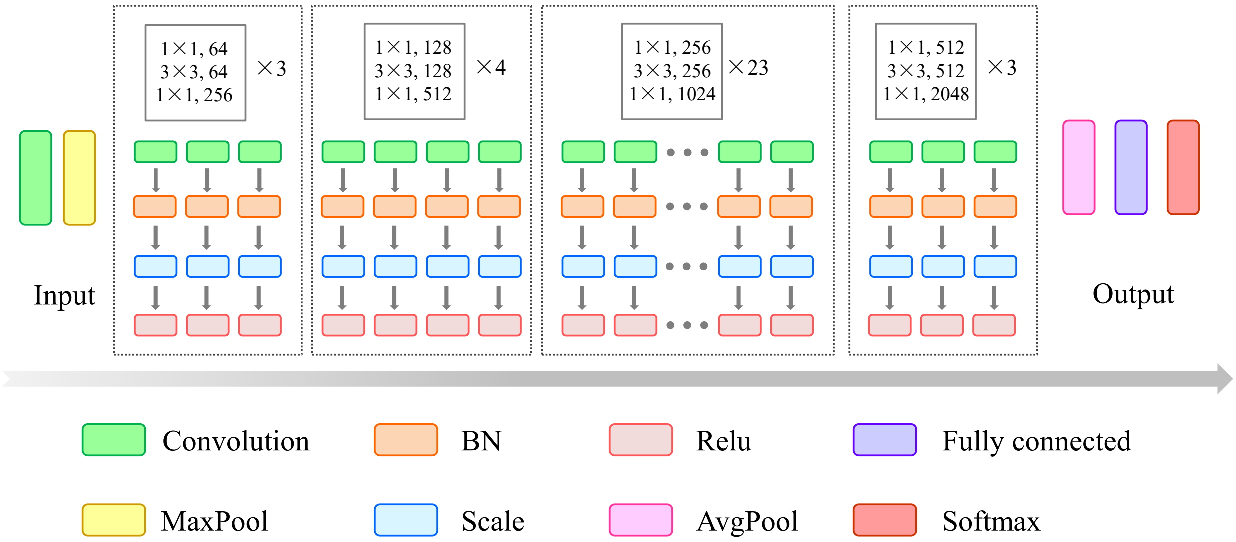

Supplementary Figure 2. Detailed architecture of the deep residual network.

Notes: MaxPool = Max Pooling; BN = Batch Normalization; Relu = Rectified Linear Units; AvgPool = Average Pooling.

The image input comprises red, green and blue (RGB) channels. The network consists of one convolution layer, 33 residual blocks (99 layers), and one fully connected layer. Each residual block includes three BNs and scale operations situated between the convolution and rectified linear units. The average pooling and Softmax classifier were applied to the last fully connected layer.

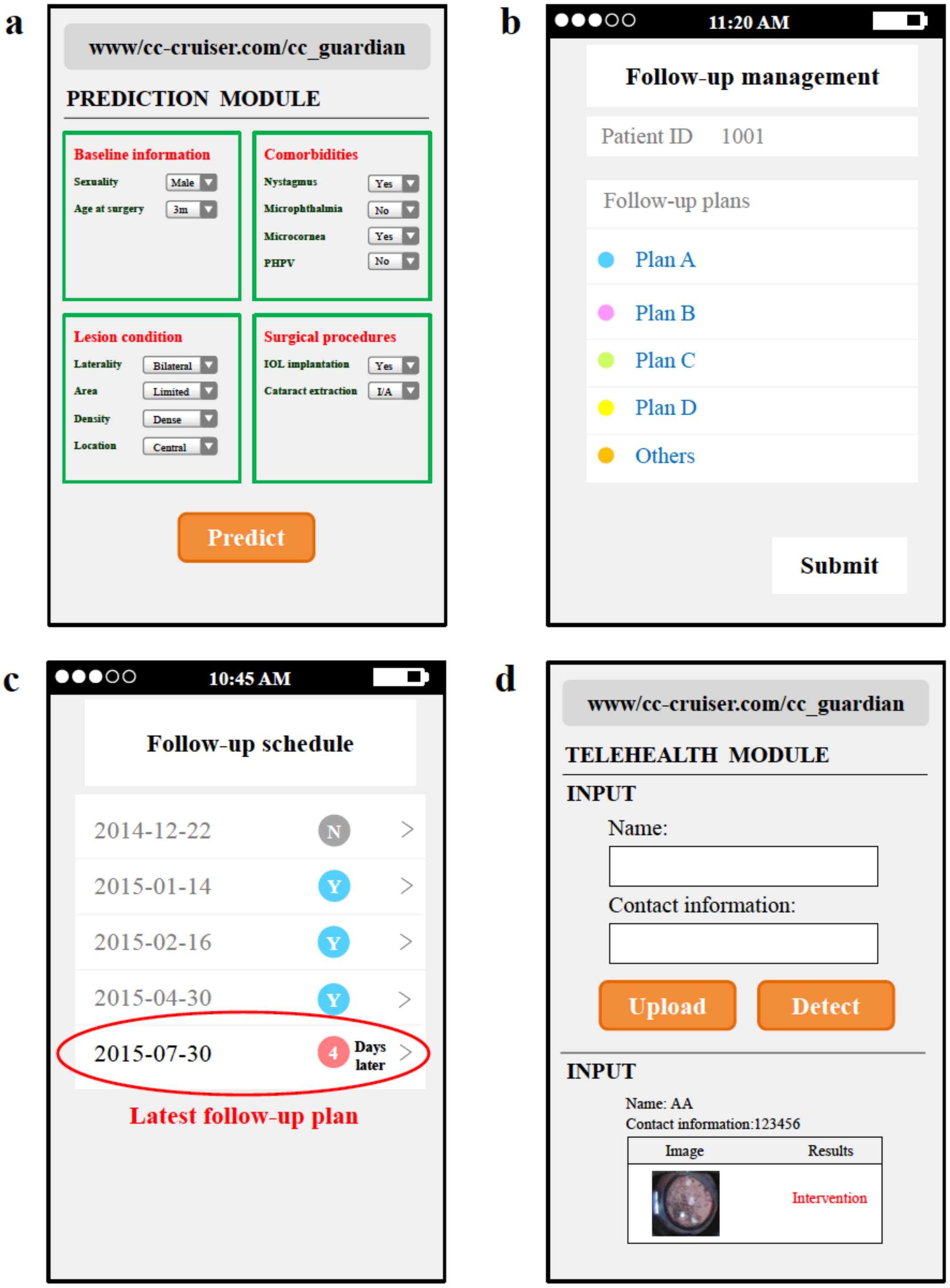

Supplementary Figure 3. User interface of the CC-Guardian website and smartphone app.

Notes: VAO = Visual Axis Opacification; IOP = Intraocular Pressure.

a. Users can login to the website and input the necessary prediction metrics to obtain risk-stratification output (occurrence of VAO and/or high IOP).

b. Doctors can assign an individual follow-up plan using the smartphone app (doctor portal).

c. Patients receive their individual follow-up schedules and can check them using the smartphone app (patient portal).

d. During the follow-up process, users can upload their follow-up examination results to the telehealth computing website and obtain a timely clinical decision.

Supplementary Tables

Supplementary Table 1. Summarized characteristics of the datasets collected from 7 centers.

Centers	Location	Total sample	VAO	High IOP
**Shenzhen Eye Hospital**	Southern China	n=25	VAO (n=12) Non-VAO (n=13)	VAO (n=10) Non-VAO (n=15)
**Beijing Tongren Hospital**	Northern China	n=16	VAO (n=8) Non-VAO (n=8)	High IOP (n=4) Normal (n=12)
**People's Hospital of Guangxi Zhuang Autonomous Region**	Southern China	n=10	VAO (n=1) Non-VAO (n=9)	High IOP (n=5) Normal (n=5)
**The Third Affiliated Hospital of Sun Yat-sen University**	Southern China	n=10	VAO (n=5) Non-VAO (n=5)	High IOP (n=1) Normal (n=9)
**Puning People's Hospital**	Southern China	n=8	VAO (n=5) Non-VAO (n=3)	High IOP (n=3) Normal (n=5)
**The Central Hospital of Wuhan**	Central China	n=6	VAO (n=2) Non-VAO (n=4)	High IOP (n=3) Normal (n=3)
**The First Affiliated Hospital of University of South China**	Southern China	n=4	VAO (n=0) Non-VAO (n=4)	High IOP (n=2) Normal (n=2)

Notes: VAO = Visual Axis Opacification; IOP = Intraocular Pressure.

Supplementary Table 2. Efficiency simulation based on different complications' prevalence.

	VAO 22%	High IOP 10%	VAO 45%	High IOP 12%	VAO 24%	High IOP 4%
**Predictive accuracy**	96.8% 30/31	100% 14/14	96.8% 61/63	94.1% 16/17	94.1% 32/34	100% 6/6
**Benefited individuals**	83.3% 25/30	92.9% 13/14	80.3% 49/61	93.8% 15/16	81.3% 26/32	83.3% 5/6
**Advanced detection (per person)**	37.6 risk days	18.7 risk days	37.4 risk days	20.2 risk days	37.5 risk days	19.9 risk days
**Saving distance (per family)**	972.2 miles (1185.4 vs. 213.2 miles, *P*<0.001)		960.0 miles (1185.4 vs. 225.4 miles, *P*<0.001)		966.2 miles (1185.4 vs. 219.2 miles, *P*<0.001)	
**Saving time (per family)**	26.4 hours (33.8 vs. 7.4 hours, *P*<0.001)		25.9 hours (33.8 vs. 7.9 hours, *P*<0.001)		26.2 hours (33.8 vs. 7.6 hours, *P*<0.001)	
**Saving expenditure (per family)**	$1395.2 ($1,791.4 vs. $396.2, *P*<0.001)		$1386.9 ($1,791.4 vs. $404.5, *P*<0.001)		$1393.0 ($1,791.4 vs. $398.4, *P*<0.001)	

Notes: VAO = Visual Axis Opacification; IOP = Intraocular Pressure.

Supplementary Table 3. Summarized characteristics of variables for the prediction module training.

Variables	Detailed description
**Gender**	Male (n=339, 57.07%)<br>Female (n=255, 42.93%)
**Age at surgery**	34.68 ± 24.69 months
**Laterality**	Bilateral (n=318, 53.54%)<br>Unilateral (n=276, 46.46%)
**Area**	Extensive (n=496, 83.50%)<br>Limited (n=98, 16.50%)
**Density**	Dense (n=440, 74.07%)<br>Non-dense (n=154, 25.93%)
**Location**	Central (n=501, 84.34%)<br>Peripheral (n=93, 15.66%)
**Nystagmus**	+ (n=145, 24.41%)<br>- (n=449, 75.59%)
**Microphthalmia**	+ (n=10, 1.68%)<br>- (n=584, 98.32%)
**Microcornea**	+ (n=9, 1.52%)<br>- (n=585, 98.48%)
**PHPV**	+ (n=7, 1.18%)<br>- (n=587, 98.82%)
**IOL implantation**	Primary (n=333, 56.06%)<br>Secondary (n=261, 43.94%)
**Surgical procedures**	I/A (n=381, 64.14%)<br>I/A+PCCC (n=17, 2.86%)<br>I/A+PCCC+A-Vit (n=196, 33.00%)
**Outcome 1**	VAO (n=279, 46.97%)<br>Non-VAO (n=315, 53.03%)
**Outcome 2**	High IOP (n=341, 57.41%)<br>Normal (n=253, 42.59%)

Summarized characteristics of variables for prediction module training are tabulated. The detailed description of each characteristic is expressed as the exact sample size with a percentage of the distribution, or the mean ± 1 standard deviation. **Notes:** PHPV = Persistent Hyperplastic Primary Vitreous; IOL = Intraocular lens; I/A = Lens Aspiration; I/A+PCCC = I/A with Posterior Continuous Curvilinear Capsulorhexis; I/A+PCCC+A-Vit = I/A+PCCC and Anterior Vitrectomy; VAO = Visual Axis Opacification; IOP = Intraocular Pressure.

Supplementary Table 4. Summarized characteristics of patients included in the self-controlled test.

Variables	Detailed description
**Gender**	Male (n=74, 52.48%)<br>Female (n=67, 47.52%)
**Age at surgery**	30.51 ± 19.87 months
**Laterality**	Bilateral (n=80, 56.74%)<br>Unilateral (n=61, 43.26%)
**Area**	Extensive (n=117, 82.98%)<br>Limited (n=24, 17.02%)
**Density**	Dense (n=108, 76.60%)<br>Non-dense (n=33, 23.40%)
**Location**	Central (n=121, 85.82%)<br>Peripheral (n=20, 14.18%)
**Nystagmus**	+ (n=37, 26.24%)<br>- (n=104, 73.76%)
**Microphthalmia**	+ (n=2, 1.42%)<br>- (n=139, 98.58%)
**Microcornea**	+ (n=3, 2.13%)<br>- (n=138, 97.87%)
**PHPV**	+ (n=2, 1.42%)<br>- (n=139, 98.58%)
**IOL implantation**	Primary (n=88, 62.41%)<br>Secondary (n=53, 37.59%)
**Surgical procedures**	I/A (n=81, 57.45%)<br>I/A+PCCC (n=3, 2.13%)<br>I/A+PCCC+A-Vit (n=57, 40.43%)
**Outcome 1**	VAO (n=93, 66.00%)<br>Non-VAO (n=48, 34.00%)
**Outcome 2**	High IOP (n=105, 74.47%)<br>Normal (n=36, .25.53%)

Summarized characteristics of variables for prediction module training are tabulated. The detailed description of each characteristic is expressed as the exact sample size with a percentage of the distribution, or the mean ± 1 standard deviation. **Notes:** PHPV = Persistent Hyperplastic Primary Vitreous; IOL = Intraocular lens; I/A = Lens Aspiration; I/A+PCCC = I/A with Posterior Continuous Curvilinear Capsulorhexis; I/A+PCCC+A-Vit = I/A+PCCC and Anterior Vitrectomy; VAO = Visual Axis Opacification; IOP = Intraocular Pressure.

Supplementary Information

Efficiency simulation of our system based on different complications' prevalence.

The prevalence of VAO and high IOP varies among studies. In our self-controlled clinical test, we included 141 patients' clinical records (93 VAO, 48 non-VAO; 105 high-IOP, 36 normal) for testing, the prevalence of which is higher than those reported by previous studies.

We applied randomly pick strategy to estimate the effects of prevalence difference. For example, VAO prevalence was settled at 22% and it will be 31 patients having VAO. We then randomly pick 31 patients from the original 93 VAO patients, and calculate the efficiency only based on those 31 patients, and repeated this process 100 times to achieve the average performance. We tried VAO prevalence at 22%, 45%, and 24%, IOP prevalence at 10%, 12%, and 4%, according to previous published papers and reviewer's suggestions.

As shown in Table S3, the prevalence difference will influence the total number of benefited individuals, but not influence the model performance, advanced time per individual, and savings per family. Detection timing is determined by predictive and detective accuracy and schedules. Saving is derived from telehealth visits; thus, if less individual having complications, the number of telehealth visits will decrease and savings will be even bigger.

Supplementary Video 1. Instructions for using the prediction-telehealth cloud platform.
