# Supplementary material for: Artificial intelligence manages congenital cataract with individualized prediction and telehealth computing
Source: NPJ Digit Med. 2020 Aug 28;3:112. doi: 10.1038/s41746-020-00319-x (PMC7455726; doi:10.1038/s41746-020-00319-x)
Supplement: Supplementary file 3 — Reporting Summary [file 41746_2020_319_MOESM3_ESM.pdf]

nature research

Corresponding author(s): Haotian Lin
Last updated by author(s): Jul 6, 2020

Reporting Summary

Nature Research wishes to improve the reproducibility of the work that we publish. This form provides structure for consistency and transparency in reporting. For further information on Nature Research policies, see our Editorial Policies and the Editorial Policy Checklist.

Statistics

For all statistical analyses, confirm that the following items are present in the figure legend, table legend, main text, or Methods section.

n/a	Confirmed	
☐	☒	The exact sample size (*n*) for each experimental group/condition, given as a discrete number and unit of measurement
☐	☒	A statement on whether measurements were taken from distinct samples or whether the same sample was measured repeatedly
☐	☒	The statistical test(s) used AND whether they are one- or two-sided<br>*Only common tests should be described solely by name; describe more complex techniques in the Methods section.*
☒	☐	A description of all covariates tested
☐	☒	A description of any assumptions or corrections, such as tests of normality and adjustment for multiple comparisons
☐	☒	A full description of the statistical parameters including central tendency (e.g. means) or other basic estimates (e.g. regression coefficient) AND variation (e.g. standard deviation) or associated estimates of uncertainty (e.g. confidence intervals)
☐	☒	For null hypothesis testing, the test statistic (e.g. *F*, *t*, *r*) with confidence intervals, effect sizes, degrees of freedom and *P* value noted<br>*Give P values as exact values whenever suitable.*
☒	☐	For Bayesian analysis, information on the choice of priors and Markov chain Monte Carlo settings
☒	☐	For hierarchical and complex designs, identification of the appropriate level for tests and full reporting of outcomes
☒	☐	Estimates of effect sizes (e.g. Cohen's *d*, Pearson's *r*), indicating how they were calculated

Our web collection on statistics for biologists contains articles on many of the points above.

Software and code

Policy information about availability of computer code

Data collection	The naive Bayes algorithm was applied for prediction module training. A stacked 101-layer residual network was utilized for telehealth module training and classification.
Data analysis	The source code for the AI agent CC-Guardian is available in Github at https://github.com/longerping/cc-guardian.

For manuscripts utilizing custom algorithms or software that are central to the research but not yet described in published literature, software must be made available to editors and reviewers. We strongly encourage code deposition in a community repository (e.g. GitHub). See the Nature Research guidelines for submitting code & software for further information.

Data

Policy information about availability of data

All manuscripts must include a data availability statement. This statement should provide the following information, where applicable:

- Accession codes, unique identifiers, or web links for publicly available datasets
- A list of figures that have associated raw data
- A description of any restrictions on data availability

The authors declare that the main data supporting the results in this study are available within the paper and its Supplementary Information. The datasets generated during the study are available for research purposes from the corresponding authors on reasonable request.

Field-specific reporting

Please select the one below that is the best fit for your research. If you are not sure, read the appropriate sections before making your selection.

☒ Life sciences ☐ Behavioural & social sciences ☐ Ecological, evolutionary & environmental sciences

For a reference copy of the document with all sections, see nature.com/documents/nr-reporting-summary-flat.pdf

Life sciences study design

All studies must disclose on these points even when the disclosure is negative.

Sample size	The training dataset included clinical records of 594 CC patients before and after surgery, and 4,881 of their follow-up images from January 2011 to December 2016.
Data exclusions	The eligible criteria of patients included diagnosis of CC, informed consent, and completed records of baseline information, lesion condition, comorbidities, surgical procedures, and complications in the first follow-up year.
Replication	We verified CC-Guardian's performance using internal and multi-resource validation. We also conducted a self-controlled clinical test to investigate the real-world efficiency of our system in terms of complication prediction, telehealth detection, and cost-efficient benefits.
Randomization	There is no randomization in our study.
Blinding	Each image was independently labeled (intervention or continued follow-up) by two licensed ophthalmologists, and a third ophthalmologist was consulted when disagreements occurred. Then, a senior ophthalmologist with over 20 years of clinical CC experience verified the labels for each image. The ophthalmologists were blind and had no access to the outcome classifications.

Reporting for specific materials, systems and methods

We require information from authors about some types of materials, experimental systems and methods used in many studies. Here, indicate whether each material, system or method listed is relevant to your study. If you are not sure if a list item applies to your research, read the appropriate section before selecting a response.

Materials & experimental systems

n/a	Involved in the study
☒	☐ Antibodies
☒	☐ Eukaryotic cell lines
☒	☐ Palaeontology and archaeology
☒	☐ Animals and other organisms
☐	☒ Human research participants
☒	☐ Clinical data
☒	☐ Dual use research of concern

Methods

n/a	Involved in the study
☒	☐ ChIP-seq
☒	☐ Flow cytometry
☒	☐ MRI-based neuroimaging

Human research participants

Policy information about studies involving human research participants

Population characteristics	Specifically, we included 141 patients' clinical records (93 VAO, 48 non-VAO; 105 high-IOP, 36 normal) for prediction module testing and 987 images (394 intervention and 593 follow-up) for telehealth module testing.
Recruitment	We retrospectively used longitudinal follow-up records from CCPMOH between January 2017 and May 2018. Informed consent was obtained from all subjects. Among 172 CC patients, 141 of which were finally included because they had complete follow-up records in all visits. All images are eligible as the inputs for the telehealth module.
Ethics oversight	Our study was approved by the centralized institutional review board (IRB) of Sun Yat-sen University. It was conducted in accordance with the Declaration of Helsinki. All included datasets were anonymized according to the Health Insurance Portability and Accountability Act Safe Harbor guidelines prior to their transfer to the study investigators.

Note that full information on the approval of the study protocol must also be provided in the manuscript.
